# Assessing research culture and capacity amongst faculty at a North American chiropractic institution: an explanatory mixed methods study

**DOI:** 10.1186/s12998-024-00558-9

**Published:** 2024-11-20

**Authors:** Carol Ann Weis, Samuel J. Howarth, Diane Grondin, Danielle Southerst, Mark Fillery, Janet D’Arcy, Christine Bradaric-Baus, Silvano Mior

**Affiliations:** 1 Canadian Memorial Chiropractic College, 6100 Leslie Street, Toronto, ON M2H 3J1 Canada; 2 Institute for Disability and Rehabilitation Research at Ontario Tech University and Canadian Memorial Chiropractic College, 2000 Simcoe Street North, Oshawa, ON L1H 7K4 Canada; 3 Institute of Health Policy, Management and Evaluation, University of Toronto, 155 College Street, 4th floor, Toronto, ON M5T 3M7 Canada

**Keywords:** Focus groups, Chiropractic, Capacity building, Evidence-based medicine, Faculty, Mentors, Qualitative research

## Abstract

**Background:**

Research enables a profession to establish its cultural authority, validate its professional roles and ensure ongoing improvement in the quality of its academic programming. Despite the clear importance of research, a mature research culture has eluded the chiropractic profession. A fostering institutional culture that enables, values, and supports research activity is essential to building research capacity. Our study aimed to collect information about the existing research capacity and culture at the Canadian Memorial Chiropractic College (CMCC) and explore the views, attitudes and experiences of faculty members regarding research.

**Methods:**

We conducted a sequential explanatory mixed methods study with quantitative priority between April and July, 2023. Quantitative data were collected using the Research Capacity and Culture (RCC) tool. Survey results guided the qualitative data collected from four faculty focus groups with varying levels of research experience. Quantitative data were analyzed using descriptive statistics by domain and stratified by research education and workload. The qualitative data were thematically analyzed and then integrated with the quantitative results to provide deeper meaning to the results.

**Results:**

The faculty survey response rate was 42% (59/144). Attributes at the organization or department level were consistently rated as either moderate or high; however, research skills at an individual level were more variable and influenced by factors such as research workload and highest research-related academic qualification. Qualitative focus group data were categorized under four themes: institutional factors, resource allocation, career pathways and personal factors. Lower scores for survey items related to mentorship, research planning and ensuring faculty research career pathways, as well as the identified workload and time-related barriers (e.g., other work roles and desire for work/life balance) for engaging in research were supported by each of the four themes. Research motivators included keeping the brain stimulated, developing skills and increasing job satisfaction.

**Conclusion:**

The quantitative and qualitative information in this study provides a baseline evaluation for RCC and identifies key factors impacting RCC at the CMCC. This information is critical for planning, developing, implementing, and evaluating future interventions to enhance research capacity. Ultimately, these efforts are aimed at maturing the research culture of the chiropractic profession.

**Supplementary Information:**

The online version contains supplementary material available at 10.1186/s12998-024-00558-9.

## Introduction

Research capacity came to the forefront for the chiropractic profession in 1996, a time that coincided with the advancement of evidence-based medicine [1,2]. The shift toward evidence-based medicine has emphasized research as an essential activity for contemporary healthcare professions to advance knowledge, provide effective and safe ways to help patients [3–7], and integrate care within the wider healthcare system [8]. Research enables a profession to establish its cultural authority, validate its professional roles and ensure ongoing improvement in the quality of its academic programming [9]. Despite the clear importance of research, in general a mature research culture has eluded the chiropractic profession; [8] however, there are institutions where such culture is maturing and growing. As “centers of advanced learning”, a fundamental role of chiropractic academic institutions is to promote scholarly activity and enhance research productivity [10]. Thus, understanding the extant research culture and capacity at chiropractic academic institutions is a necessary precursor to determining strategies that would mature the profession’s research culture [10]. 

We defined research capacity as the ability and skill of individuals and institutions to perform high quality research, which is adapted from Trostle’s definition for “research capacity building” [11]. Here, “ability” refers to both the aptitude of people and the infrastructure at institutions for conducting research. Research culture is defined by the Royal Society, United Kingdom, as “the behaviors, values, expectations, attitudes and norms of our research communities” [12,13]. Commonly, this definition of research culture focuses on groups such as academic institutions and departments, but can also include professional societies, associations and networks. A supportive research culture can enable research capacity, whereas an unsupportive or negative research culture can hinder research capacity.

Recent scholarly attention has focused mainly on building research capacity in the chiropractic profession (including specialties) or describing programs aimed at developing networks of researchers [3,5,8,10,14]. A recent scoping review revealed that only two published studies have focused on research capacity at chiropractic academic institutions since 2010 [12]. This suggests that the existing knowledge of research capacity at chiropractic academic institutions is likely outdated. Anderson and colleagues qualitatively grouped findings from their scoping review under five themes that were comprised of subthemes related to barriers and facilitators to chiropractic faculty research. Subthemes such as academic rank and educational background, motivation, autonomy, attitudes toward research by leadership, financial incentives, release time and mentorship were identified by Anderson and colleagues as facilitators of research capacity. These sub-themes coincide with findings from studies on research culture and capacity in allied, primary and community healthcare services [7,15,16], which also overlap with the model of a productive research environment described by Bland and colleagues [17]. Thus, it appears that facilitators of research capacity and the development of a positive research culture for chiropractic academic institutions are similar to those in other healthcare professions. Interestingly, there have not been any published trials evaluating strategies for enhancing research capacity at chiropractic academic institutions [12]. 

The Canadian Memorial Chiropractic College (CMCC) has historically valued research and, according to a bibliometric analysis of the Web of Science Core Collection, has published the most research on low back pain and manual therapy between 2000 and 2023; [18,19] however, other bibliometric evidence suggests that research productivity (defined by the number of publications per year) at the CMCC has plateaued [20]. It is unclear whether this plateau is an indication that faculty have maximized their current level of productivity or if there has been a change in capacity or culture. Thus, targeted efforts addressing potential changes in research culture and sustainable research capacity at the CMCC would benefit from baseline information. Therefore, the aim of the current investigation was to collect information about the extant RCC at the CMCC and to explore the views, attitudes and experiences of faculty members regarding research.

## Methods

A sequential explanatory mixed methods design with quantitative priority [21] was used to evaluate RCC at the CMCC. Mixed methods research allows for the collection and analysis of both qualitative and quantitative data and their integration, drawing on the strengths of both approaches [22]. In this explanatory design, we first conducted the quantitative data collection and analysis, and the results then informed the follow-up qualitative phase of the study. It was determined a priori that the quantitative data would be prioritized in our analysis but would not be sufficient to address the complex and multifaceted issues underpinning RCC [23]. Therefore, the qualitative data was used to provide a deeper understanding of the quantitative survey findings, as well capturing the experiences and perspectives of faculty related to research.

Ethical approval for this study was granted by the Research Ethics Board at the CMCC (REB #: 2302B02). Our study was reported according to the Good Reporting of a Mixed Methods Study (GRAMMS) checklist (Additional file 1) [24]. 

### Setting, participants & recruitment

In its June 2022 Strategic Plan, the CMCC committed to focusing its research efforts within five different streams. The five streams are used to categorize faculty’s research area of interest and include the biological basis of musculoskeletal injury and manual therapies, clinical and health services research, education in health care, health and wellness and knowledge translation and health policy. The Division of Research and Innovation at the CMCC is led by a member of the institution’s executive team and administratively supported by four directors overseeing their respective departments, supported by a research services administrator. CMCC is a unionized environment, where full- and part-time unionized faculty members are provided an annual workload that may include teaching, administrative duties and/or research. Regardless of workload, research productivity (i.e., grants applied for and received, peer-reviewed publications, conference presentations) is required by the Collective Agreement for promotion in academic rank. Unionized faculty without an allocated research workload have the opportunity to engage in research through various modes, including a competitive application process for research hours and project funding [25,26]. Regardless of their workload requirements, involvement in research or academic rank at the institution, all the faculty at the CMCC were invited via their institutional e-mail to complete an online questionnaire. Participation was voluntary and there was no financial compensation for completing the survey or participating in the focus groups.

Participants were recruited for the quantitative component using a modified tailored design method [27] from April until May 2023. Awareness was raised through a pre-survey email sent the week preceding the survey launch by the Vice-President, Academic and the Faculty Union President. To maximize the response rate, a series of three reminder emails were sent to the faculty over five weeks. Survey emails included messages of support from “influencers”, individuals considered mentors or leaders within their department.

Purposive sampling was used to recruit focus group participants. First, participants who completed the survey were invited to participate in one of four focus groups to provide an in-depth exploration of the survey results. Second, e-mail invitations were sent to faculty members with various levels of research experience to ensure that each focus group was well represented. Those who agreed to participate in a focus group were selected using maximum variation sampling to create groups of 8–10 faculty members balanced with respect to gender, department, role, and duration of employment. Focus groups were conducted from June to July 2023.

A research assistant screened respondents who had confirmed interest in participating in one of four focus groups based on their answers to screening questions included in the survey. Group 1 included faculty who were very active in conducting research. Group 2 included faculty who had some experience in research or who were interested in conducting research. Group 3 consisted of faculty who had very little or no experience in conducting research and/or who were not interested in conducting research. Group 4 consisted of departmental administrative managers with varying levels of research experience. We stratified focus groups into four distinct and homogeneous groups composed of members with similar backgrounds thereby avoiding potential power or role differentials and allowing for a permissive and nonthreatening setting [28]. 

### Data collection

In consideration of our mixed methods design, we conceptualized that data collection and synthesis could be captured within the theoretical stance of pragmatism. Pragmatism enables the use of multiple methods of data collection to address the research aims whilst focusing on the practical implications of the research [29]. 

#### Quantitative component

A cross-sectional survey was used to collect quantitative data. The survey was administered using an internet-based web interface tool, SurveyMonkey^®^ (San Mateo, CA, USA), which participants accessed through computers or mobile devices. A survey invitation and a uniform resource locator (URL) link to the survey were emailed to all potential participants. The survey URL directed participants to the first page of the online survey that provided study information, including the purpose, description of their involvement, risks and benefits, and voluntary nature of participation in the study. Participants confirmed their consent by selecting to “*agree*” to continue with the survey.

The survey comprised two sections. The first section asked participants to provide demographic information, including age, gender, professional qualifications, enrollment in higher degree studies or other professional development related to research, duration of employment, primary department of appointment, whether their workload included specific research time and percentage, if research hours had been applied for, publications in peer-reviewed journals, whether they had obtained internal or external research funding and any presentations at scientific conferences. Quantitative data were obtained in the second section using questions from the RCC tool [30]. 

The RCC tool is a validated questionnaire to quantitatively evaluate research culture and capacity at the organizational, team and individual levels [30,31]. To assess the skill or success in research at each level 52 items are included; with 18 items at the organizational level, 19 items at the team level and 15 items at the individual level. Each item was scored on a 10-point Likert scale, with 10 being the highest possible level of skill or success [30,31]. The respondents were also provided an “unsure” option for each question. The RCC tool also asked respondents to select applicable barriers and motivators to participate in research from a list of 18 barriers and 18 motivators. Additional barriers and motivators were provided by respondents, if desired. Measurement properties suggest good internal consistency for each domain of organization, team and individual (α = 0.95, 0.96 and 0.96 respectively) and factor loading (ranging between 0.58 and 0.89, 0.65–0.89 and 0.59–0.93 respectively). Additionally, there is adequate test-retest reliability, with intraclass correlations of 0.77 (organizational), 0.83 (team) and 0.82 (individual) [30]. 

Minor modifications were made to the RCC tool to reflect the unique organizational and faculty structure at the CMCC. In the ‘Information about your role’ subsection within the demographics, options for department and role were changed specific to CMCC. A question was added for participants to identify their research stream (if applicable) as described in the institution’s Strategic Plan. Rather than asking about success and skill at the team level, the wording was changed to reflect the “departmental” level, consistent with the CMCC’s organizational structure. We added clarification to some questions where we felt ambiguity could compromise the interpretation of the question and made them more specific to our environment, e.g., staff to faculty. Demographic questions related to age, gender, duration of employment, academic and clinical credentials (including post-graduate residency programs which require research work) and extent of research involvement at the CMCC were also added. The final question of the survey asked participants if they would be interested in participating in the focus group portion of the study. If they answered “Yes” to this question, participants were asked to leave their name and email for future correspondence regarding the focus group. If they answered “No” to the question, they were thanked for their participation.

Finally, the modified RCC tool was pilot tested to ensure comprehensibility. We distributed the modified tool to two researchers with survey experience associated with two different chiropractic institutions. These researchers reviewed the survey for grammatical errors, relevance and clarity of the questions. Findings from the quantitative component were used to inform the qualitative phase of the study.

#### Qualitative component

We used focus groups to explore survey results and understand faculty members’ experiences participating in research at the CMCC. Focus groups are a form of group interview that aims to create an understanding of the social dynamics and interactions between participants, through a collection of verbal and observational data [32]. They are also useful for exploring people’s knowledge and experiences [28,32]. A semi-structured focus group interview guide was developed based on the survey results and included open-ended questions and probes to explore individuals’ perspectives and experiences regarding research at the CMCC (Additional File 2).

Focus group sessions were facilitated by a trained qualitative researcher. This individual was known to many participants as colleague and research faculty at the CMCC who had been away from direct interactions with the CMCC community for 2–3 years immediately preceding the sessions. This permitted the individual to have general knowledge about research at the CMCC but be less aware of any recent specific comments that may be raised by the participants. The sessions were conducted and recorded over Zoom (San Jose, CA, USA) to enable participation of part-time faculty and clinical faculty who have other external commitments that could limit their in-person participation. Focus group sessions lasted 90 to 120 min. A research assistant oversaw the session recording, took notes and observed participant interactions and occasionally probed for clarification or deeper exploration where indicated. The assistant was not a CMCC faculty member or employee, thereby facilitating neutrality. However, they did attend the CMCC as an undergraduate and graduate student and, thus were familiar with some participants. To acknowledge and minimize the influence that pre-existing relationships may have had on the focus groups [29], the facilitator and assistant debriefed following each session. The facilitator engaged in reflexivity, noting any assumptions based on previous background and experiences and discussing any concerns at team meetings [33]. 

### Data storage

Survey data were downloaded from SurveyMonkey^®^ in a CSV format and uploaded as an Excel file. Responses from each participant were de-identified and assigned a unique respondent ID assigned by SurveyMonkey^®^. To ensure anonymity of the participant responses, an independent research assistant removed the IP addresses and any email addresses from the data set prior to study personnel analyzing the data.

For the qualitative data, only the audio portion of the recording was retained for analysis. The audio recording was transcribed verbatim by an individual not directly associated with the project. During the transcription process all names were replaced by pseudonyms to ensure anonymity. The accuracy of transcription and removal of identifying features were performed by double-checking the transcripts with the recorded session. Digital audio files generated from the recordings and transcripts of the focus groups were password protected and stored on CMCC secure servers.

### Data analysis

All quantitative data analyses were completed using Excel (Microsoft Corp., Redmond, WA, USA). Sample demographics are reported descriptively using means, standard deviations, frequencies and percentages.

All items in each domain were reported on an ordinal scale of 1–10. The median and interquartile range were calculated, and the number of “unsure” responses was counted for each item in the three domains of the RCC tool. The median scores were interpreted as high (median score *≥* 7), moderate (median score 4–6) or low (median score < 4).^[7, 15^.

Two stratifications were applied to the responses from the RCC tool. These stratifications were by each participant’s highest research-related qualification and by whether their workload included research. The decision to apply these stratifications was made *post hoc* as part of our explanatory analysis of the data and after having confirmed a relatively equal distribution of respondents for each level of each stratification. Each of these stratifications has been previously reported in other studies as contributors to either personal research skills or enhanced research culture and capacity [7,34]. 

The focus group data were thematically analyzed as described by Braun and Clarke [35]. Coders used both deductive (informed by quantitative data and research) and inductive (emergent categories) codes that were summarily described in the codebook. Each transcript was read, reviewed and then line-by-line independently coded by two different individuals on the research team. Coders then met to engage in debriefing session to discuss their findings and reach consensus on any discrepancies. Themes were developed by reading through the data to identify patterned responses or meanings, named and defined. Themes that arose iteratively from the data were discussed, reviewed for their coherence with individual coded extracts, and collaboratively agreed to by the full research team. This process enhanced the trustworthiness of the analysis [36]. 

Finally, to assess the trustworthiness of, and confirm our interpretation the data reflected participants’ voices, we triangulated our results and assessed the authenticity of our analysis [37] by presenting our preliminary findings at an open internal research meeting attended by faculty. We summarized the survey and qualitative results, shared our interpretation of the data, and then sought additional feedback from attendees. Topics for small group discussion included themes that arose from the study and study quotations that pertained to one of the four topic areas, namely education/training, mentorship, communication, and student engagement. We then asked for additional thoughts, suggested opportunities and solutions to identified barriers. In addition to these meetings, the research team debriefed throughout the process and bracketed (suspending one’s personal understanding on a subject [29]) themselves when discussing results and meeting outcomes.

## Results

### Participants

Response rate for the questionnaire was 42% (61/144). The work and research characteristics of the respondents can be found in Tables 1 and 2, respectively. Approximately 47% (28/59) of respondents were part-time employees. The average duration of employment for respondents was 14 years. 64% (38/59) of respondents did not have research time as part of their annual workload; however, 29% (11/38) of these participants reported having applied for research hours through an internal competitive process. Accordingly, 74% (43/58) of respondents reported that research was not part of their job description. 36% (21/59) of respondents identified clinical and health services research as their primary area of interest. Almost half, 46% (26/57), of participants reported being named as an investigator on an external grant and 60% (26/43) of participants were an author on 1–10 publications.

**Table 1:** Demographics of the respondents

**Age**, ***n* = 59**	Avg 48.1 (14.3)	**Rank**, ***n* = 59**	
**Gender**, ***n* = 61**		Tutor	5
Man	36	Instructor	15
Woman	23	Assistant professor	14
Non-binary or gender fluid	2	Associate professor	11
**Years of employment**, ***n* = 58**	Avg 14.3 (9.7)	Professor	6
Under 1 yr	1	Other	4
1–5	10	Prefer not to answer	4
6–10	13	**Personal qualifications (highest)**, *n* = 61*	
11–15	16	Undergraduate	
16–20	7	Professional degree (i.e., DC)	
21–25	3	Fellowship/Residency	
26–30	2	Postgraduate (i.e., Masters)	
31+	6	PhD	
**Employment Status**, ***n* = 59**		Certificate/Diplomate	
Full-time	24	Prefer not to answer	
Part-time	28	More than one degree	40
Staff	1		
Prefer not to answer	6	*8 individuals currently enrolled in higher degrees	

**Table 2:** Work- and research-related activities

**Primary research stream**, ***n* = 59**		**Research activities in role description**, ***n* = 58**	
Biological basis of musculoskeletal injuries & manual therapies	6	Yes	14
Clinical and health services research	21	No	43
Education in health care	8	Prefer not to answer	1
Health and wellness	4	Awarded research hours through internal competition, *n* = 11	
Knowledge translation and health policy	3	Yes	11
Unsure	4	No	29
Not applicable	9	Prefer not to answer	4
Prefer not to answer	4		
**Workload includes research**, ***n* = 59**		Applied for research hours (if not workload), *n* = 44	
Yes	15	Yes	11
No	38	No	29
Prefer not to answer	6	Prefer not to answer	4
**Provisions made (if research in role)**, ***n* = 13**		**Authorship while at [ACADEMIC INSTIUTION]**, *n* = 57	
Time	7	Yes	43
Software	6	No	13
Training	6	Prefer not to answer	1
Library access	6	**Number of publications**, ***n* = 43**	
Research supervisions	5	1–10	26
Research funds	5	11–20	7
Administrative support	5	21–30	3
Other	2	31–40	4
		41+	2
		unsure	1
**Investigator on grant**, ***n* = 57**		**Interested in conducting research at** **[ACADEMIC INSTIUTION]**, ***n* = 23**	
Yes	26	Yes	19
No	28	No	2
Prefer not to answer	3	Prefer not to answer	2

### Survey

#### Organization research capacity

Summary data for the organization section of the survey is presented in Table 3. All attributes were rated as either moderate or high. Promotion of evidence based practice, 9 [7–10], and support for peer-reviewed publication, 9 [7–10], were the most highly rated attributes. Ensuring career pathways for research, 5 [3–7], and having consumers involved in research, 5 [3–7] were the lowest rated attributes.

**Table 3:** Organization level median scores and inter-quartile ranges (IQR). Items were scored on a 10-point likert scale*. *N* = 55

Item Description	Scored Responses (*n*)	Unsure (*n*)	Median (/10)	IQR
a. Has adequate resources to support faculty research training	47	8	6	4–8
b. Has funds, equipment or administrative staff to support research activities	48	7	6	4–8
c. Has a plan or policy for research development	46	9	7	4–8
d. Has senior managers that support research	52	3	8	5–9
e. Ensures faculty career pathways are available in research.	47	8	5	3–7
f. Ensures organization planning is guided by evidence (for example, measures research productivity or performance, decisions informed by organizational research strengths and opportunities in the external environment)	45	10	7	4–8
g. Has consumers involved in research	33	22	5	3–7
h. Accesses external funding for research	45	10	7	5–8
i. Promotes clinical practice based on evidence	51	4	9	7–10
j. Encourages research activities relevant to practice	51	4	7	5–9
k. Has software programs for analyzing research data	34	21	8	6–9
l. Has mechanisms to monitor research quality	34	21	7	4–9
m. Has identified experts accessible for research advice	44	11	8	5–9
n. Supports a multi-disciplinary approach to research	43	12	7	4–9
o. Supports inter-departmental research collaboration	43	12	7	4–9
p. Has regular forums/bulletins to present research findings	50	5	6	3–8
q. Engages external partners (e.g., universities, chiropractic programs, other professional programs) in research	45	10	7	5–8
r. Supports applications for research scholarships/degrees	45	10	7	4–9
s. Supports the peer-reviewed publication of research	48	7	9	7–10

*Likert scale: 1 = no success/skill and 10 = highest possible success/skill

On average, the percentage of “unsure” responses was 18% (10/55) at the organization level. The attribute of having mechanisms to monitor research quality was rated high, but 38% (21/55) of respondents responded “unsure”. The highest percentage of “unsure” responses, 40% (22/55), occurred for the attribute of having consumers involved in research.

#### Department research capacity

Summary data for the department section of the survey is presented in Table 4. All attributes were rated as either moderate or high. Support for peer-reviewed publication of research, 9 [7–10], was the highest rated attribute. The lowest rated item was having incentives for mentoring activities, 5 [2–7].

**Table 4:** Departmental level median scores and inter-quartile ranges (IQR). Items were scored on a 10-point likert scale*. *N* = 45

Item Description	Scored Responses (*n*)	Unsure (*n*)	Median (/10)	IQR
a. Has adequate resources to support faculty research training	38	7	7	2–8
b. Has funds, equipment or administrative staff to support research activities	36	9	7	2–8
c. Does department-level planning for research development	37	8	6	2–8
d. Ensures faculty involvement in developing that plan	36	9	6	2–8
e. Has departmental leaders that support research	40	5	8	6–9
f. Provides opportunities to get involved in research	39	6	7	4–9
g. Does planning guided by evidence (e.g., measures research productivity or performance, decisions informed by departmental research strengths and opportunities in the external environment)	34	11	6	2–9
h. Has consumers involved in research	27	18	6	2–8
i. Has applied for external funding for research	30	15	7	3–8
j. Conducts research activities relevant to practice	35	10	8	6–9
k. Supports application for research scholarships/degrees	36	9	8	6–9
l. Has mechanisms to monitor research quality	30	15	6	2–8
m. Has identified experts accessible for research advice	34	11	8	5–9
n. Disseminates research results at research forums/seminars	39	6	8	6–9
o. Supports a multi-disciplinary approach to research	38	7	7	4–9
p. Supports inter-departmental research collaboration	35	10	7	4–9
q. Has incentives and support for mentoring activities	37	8	5	2–7
r. Has external partners in research (e.g., universities/colleges, professional organizations, industry)	37	8	7	5–8
s. Supports the peer-reviewed publication of research	37	8	9	7–10
t. Has software available to support research activities	29	16	7	2–8

*Likert scale: 1 = no success/skill and 10 = highest possible success/skill

On average, the percentage of “unsure” responses was 22% (10/45) at the department level. The attribute of having software for data analyses was rated high, but 36% (16/45) of respondents responded “unsure”. The highest percentage of “unsure” responses, 40% (18/45), occurred for the attribute of having consumers involved in research.

#### Individual research capacity

Summary data for the individual section of the survey is presented in Table 5. The respondents were most confident with finding literature, 8 [7–9], and appraising literature, 8 [6–9]. Perceived skill level was lowest for writing research reports, 5 [3–9], securing research funding, 5 [2–7], designing questionnaires, 5 [3–8], and providing advice to less experienced researchers, 5 [3–8]. The average percentage of “unsure” responses across all elements of the individual section for the survey was 2% (1/44).

**Table 5:** Individual level median scores and interquartile ranges (IQR). Items were scored on a 10-point likert scale*. *N* = 44

Item Description	Scored Responses (*n*)	Unsure (*n*)	Median (/10)	IQR
a. Finding relevant literature	44	0	8	7–9
b. Critically appraising the literature	44	0	8	6–9
c. Using a computer referencing system (e.g., Endnote)	43	1	6	4–8
d. Writing and implementing a research protocol (e.g., creating a research question, study design, plan for data collection/analysis)	43	1	7	5–8
e. Securing research funding	42	2	5	2–7
f. Submitting a successful application to the Research Ethics Board	44	0	7	4–9
g. Designing questionnaires	40	4	5	3–8
h. Collecting data (e.g., surveys, interviews, standardized instruments, using laboratory equipment)	43	1	6	4–8
i. Using computer data management systems (e.g., RedCap, SurveyMonkey, Distiller, EPPI Reviewer, RevMan, Excel)	40	4	6	2–8
j. Analyzing qualitative research data	41	3	6	3–7
k. Analyzing quantitative research data	42	2	7	4–8
l. Writing a research report (e.g., report to a funding agency or stakeholders)	43	1	5	3–9
m. Selecting a journal for publication	43	1	7	5–8
n. Writing for publication in peer-reviewed journals	44	0	7	5–9
o. Providing advice to less experienced researchers	43	1	5	3–8

*Likert Scale: 1 = no success/skill and 10 = highest possible success/skill

#### Barriers/motivators

Table 6 outlines the individual motivators and barriers related to research culture and capacity. The top three reasons that respondents cited as barriers to becoming involved in research included lack of time, 70% (31/44), other work roles taking priority, 57% (25/44), and the desire for work/life balance, 45% (20/44). The top three reasons respondents were motivated to stay engaged in research included maintaining brain stimulation, 68% (30/44), developing skills, 64% (28/44), and increasing job satisfaction, 48% (21/44).

**Table 6:** Personal barriers and motivators, *n* = 44

Personal barriers*		Personal motivators*	
Lack of time	31	To keep the brain stimulated	30
Other work roles take priority	25	Develop skills	28
Desire for work/life balance	20	Increased job satisfaction	21
Lack of funds for research	19	Problem identified that needs changing	18
Other personal commitments	16	Career advancement	17
Lack of administrative support	14	Increased credibility	15
Lack of coordinated approach to research	14	Promotion in rank	14
Lack of research skills	14	Desire to prove a theory/hunch	14
Lack of support from management	10	Opportunities to participate at own level	12
isolation	9	Colleagues doing research	11
Intimidated by fear of getting it wrong	6	Mentors available to supervise	11
Lack of access to equipment for research	4	Research encouraged by managers	10
Lack of software for research	4	Study or research scholarships available	8
Intimidated by research language	4	Dedicated time for research	7
Lack of suitable backfill	3	Grant funds – Links to universities	6
Lack of library/internet access	0	Forms part of post-graduated study	5
Not interested in research	0	Research written into role descriptions	4
Other	6	Other	7
Prefer not to answer	1	Prefer not to answer	0

*Respondents could choose more than one response

#### Stratification by research workload

**Fig. 1:**
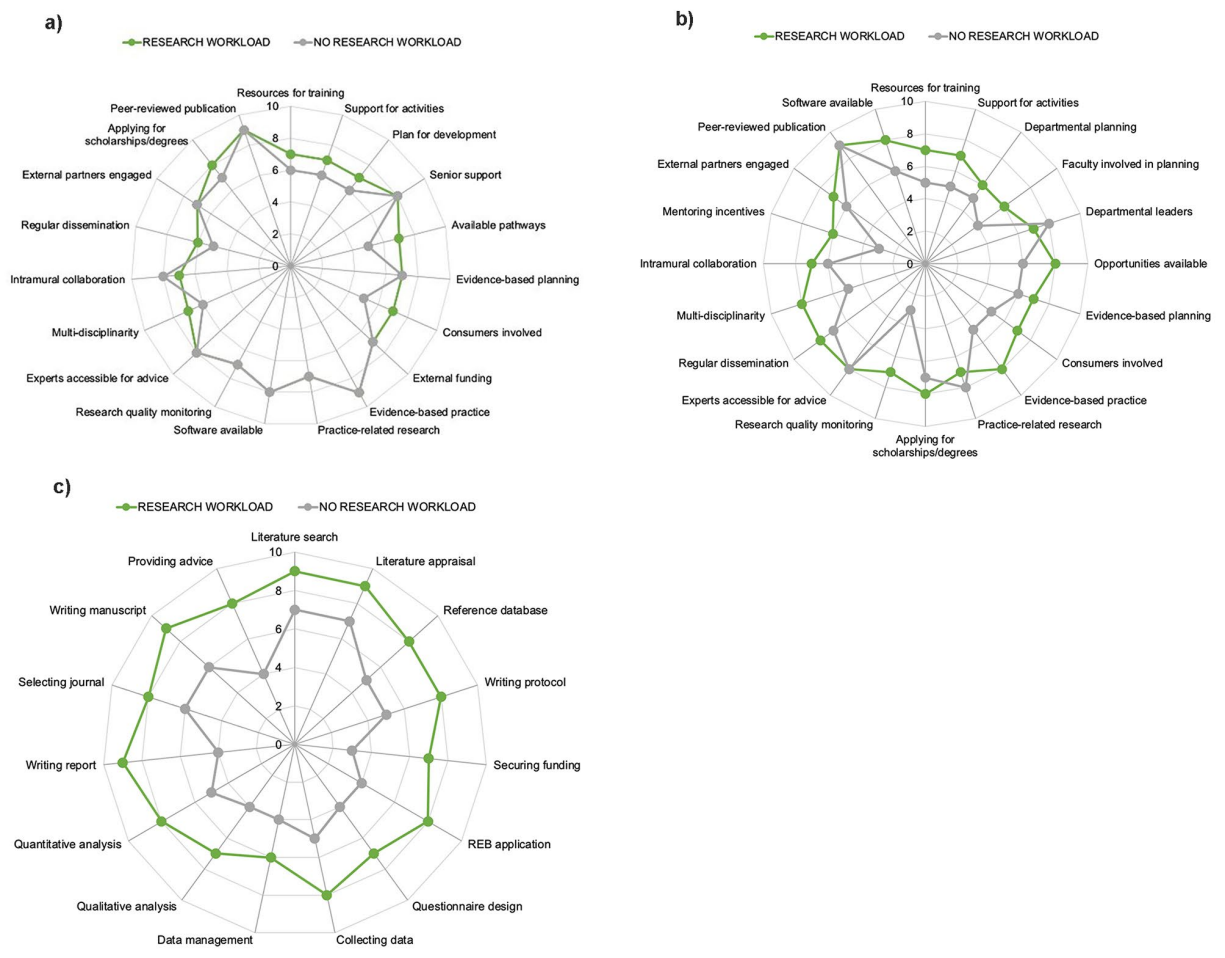
Stratification of the RCC tool by research workload

There were no substantial differences in skill/success ratings at the organization level between respondents who reported having research as part of their workload and those who did not have research as part of their workload (Fig. 1A). At the department level, the largest discrepancies between these two subgroups of respondents were related to incentives for mentoring and knowing whether mechanisms exist for monitoring research quality (Fig. 1B). The median score for respondents who did not identify as having a research workload was lower in both cases. Similarly, respondents without research workload were less confident in their own research-related skills (Fig. 1C).

#### Stratification by highest research-related academic qualification

**Fig. 2:**
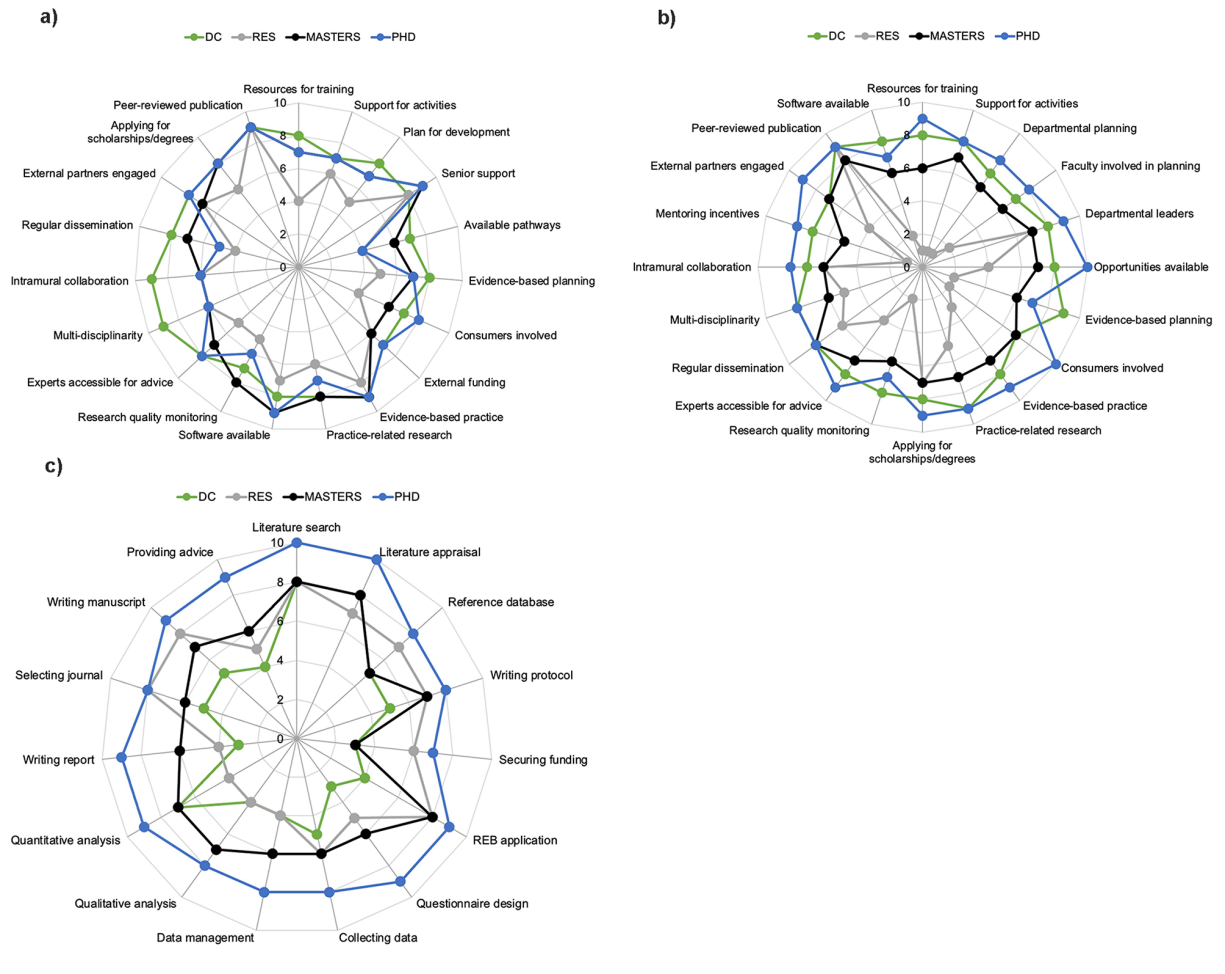
Stratification of the RCC Tool by highest research-related academic qualification

At the organization level, respondents rated all attributes to a similar extent regardless of their highest research-related academic qualification (Fig. 2A). Those who had completed a residency program (i.e., fellowship designation) as their highest research-related academic qualification rated many of the items at the department level as low (Fig. 2B). Respondents with a PhD had the highest rating of self-perceived research skills across all items in the individual section of the survey (Fig. 2C).

### Focus group

**Fig. 3:**
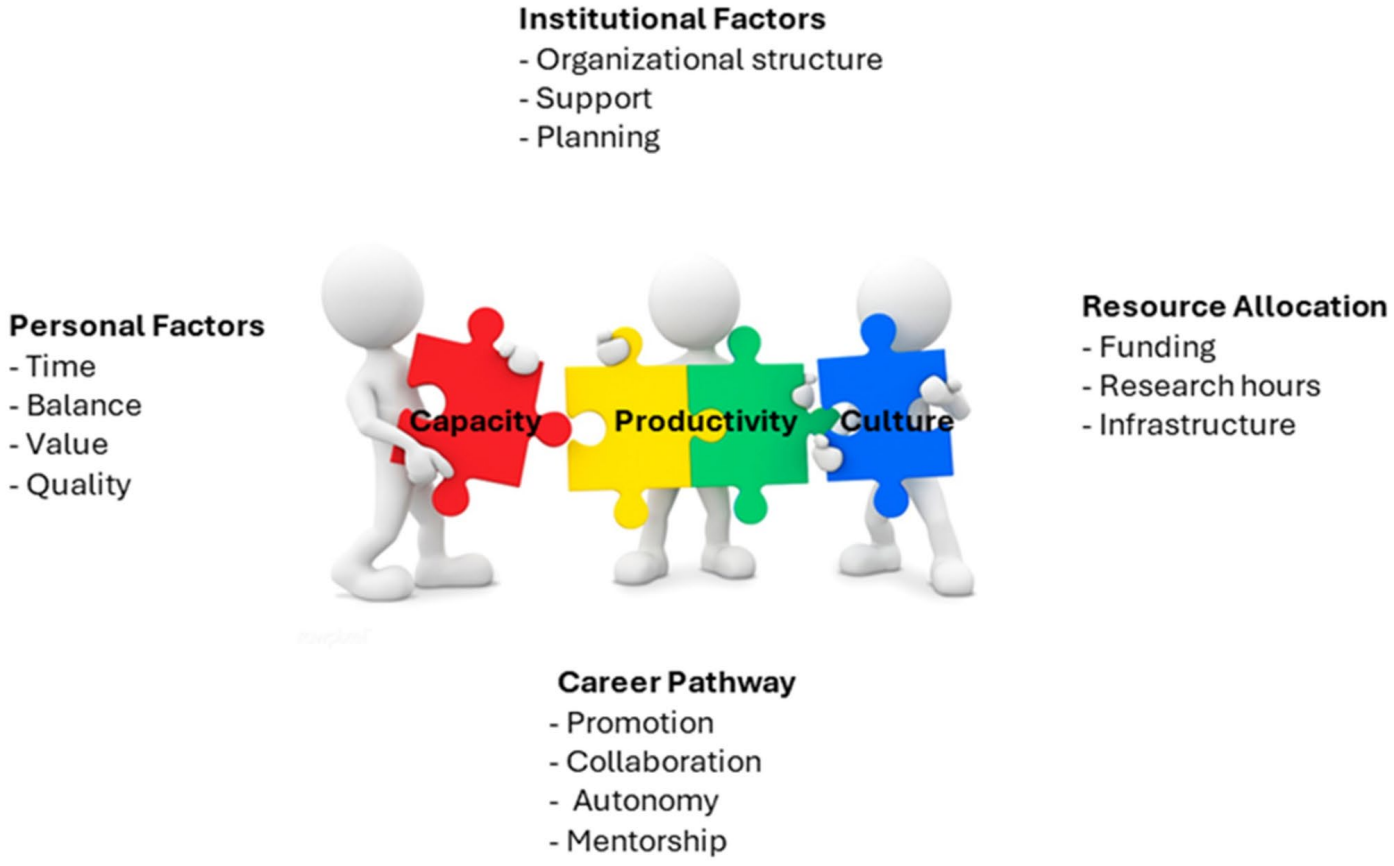
Summary of emergent themes and related categories

Four themes and their associated categories that emerged from the data were identified. The four main themes included personal factors, career pathway, resource allocation and institutional factors (Fig. 3). The themes resonated with each focus group; however, the degree of agreement or relevance differed depending upon the level of research experience or administrative duties among the focus group participants.

#### Personal factors

The theme “personal factors” captured participants’ personal experiences and issues arising in the conduct of research. Sub-themes included perception of value of work, differentiating between the quality and quantity of research work, allocated time for research, and balancing work and personal time. The perception of valuing research was identified as a motivator for participating and conducting research and was linked to the attribution of the importance of the work but also advancing the science. Highlighting such value was an appreciation of the quality rather than the quantity of research, as noted by an administrator participant.

*“I’m all in for getting more researchers*, *as long as we’re getting quality research….I’m not about quantity.”* (FG4: 725–730).

However, one personal factor identified as an issue by all participants was time allocated for conducting research, particularly among those with limited research experience who typically would not have research hours included in their workload, as aptly described by two participants:

*“…if I had to put my finger on one issue that I think holds people back from being involved in research is the time allocation.”* FG3:335–337.

and

*“Time constraints for sure are a major problem*, *when we wear as many hats as we do. It’s very hard to focus on research and give it your all*, *unless you work on Saturdays and Sundays*, *as well and I think we should be past that.”* FG2: 199–202.

Due to the requirements for promotion, faculty members do conduct or participate in research even if workload hours are not allocated. However, participants with little interest in conducting research struggled with balancing and prioritizing the demands of work and personal life, often leaving research work aside in preference to their teaching duties:

*“After I’m here 6:30 in the morning*, *I get home*, *5:30 at night*, *and then I am asked to do research after that period of time? Or do I neglect some of my duties at school and try to*, *double task by integrating research while I’m supposed to be teaching? So*, *it’s a tough balance.”* FG3: 353–357.

#### Career pathway

The second identified theme related to participants viewing research as a “career pathway” to professional advancement and promotion and a key mechanism to building capacity in research. The subthemes included promotion, collaboration, autonomy and mentorship. Participants acknowledged that promotion was a key driver to participating in and contributing to research productivity; however, this evoked emotional and contentious responses by participants in each focus group. Opinions about promotion driving capacity and productivity differed depending upon the level of research experience and their faculty role or position but appeared to impact mostly those with little experience or interest in research, leaving them feeling discouraged.

*“I think*, *clear incentives of doing research needs to be clearly stated except for the promotion part. I think I’m more discouraged as I learn more and more about the hardness of doing the research and the barriers that I need to actually go through.”* FG3:1035–1038.

Others were more pragmatic about the role promotion plays in advancing research; an important role consistent with the reality found in academic institutions:

*“And for each one of the academic institutions that I’ve taught and done research in …[research was] always tied to promotion*, *as we have here*, *research*, *service*, *and teaching. It is important for any kind of academic institution. This would be something that would be very important.”* FG1: 855–860.

and

*“And as has been mentioned a few times*, *[doing research] like external motivation is a big thing*, *as well trying to work for promotion.”* FG2:661–663.

However, participants in the administrative group (Group 4) were more cautious in their positive attribution of promotion being used as a leverage to further research, suggesting doing so may lead to higher productivity but also impact the quality of work being produced:

*“Again*, *it comes down to*, *I guess what the institution*, *what the administration wants to use as motivation to make people do research. If you’re asking for research to be done proficiently*, *effectively*, *and to a scholarly level*, *and that’s not to say that everybody that’s looking for promotion is not doing that*, *but I do think that you get a very different quality of research from true researchers that do it for the sake of doing research.”* FG4: 461–465.

Despite the inherent issues underlying the direct and indirect consequences of promotions driving culture and capacity, participants in all focus groups did appreciate the autonomy and freedom to determine their own line of inquiry and research. Although the institution has strategically identified streams of research, they are sufficiently broadly defined to allow faculty to pursue their areas of scholarly interest. In addition, participants identified the benefits of collaboration and networking as mechanisms to further their career path by contributing to their research productivity, while offsetting their personal time constraints or gaps in knowledge or skills. This was poignantly described by focus group participants with no experience in research:

*“I’m very sure if we change that [research] culture*, *that mindset within CMCC and foster that culture. So many people would be interested in doing research and we won’t have that shortage of hands-on deck to help and collaborate together*, *and it’s not such a time constraint for everybody. I think that’s something that really should be talked about and discussed strategically with more focus groups.”* FG3: 543–548.

Participants in all focus groups described the importance of mentorship in assisting with developing faculty research career pathways. They described how mentorship could encourage faculty to participate and conduct research, given that many were not formally trained. Some shared ideas and positive experiences of being mentored, whilst others had different experiences leading to feelings of frustration and intimidation, such as:

*“So*, *you know*, *when I find one person and they were very helpful and encouraging*, *and that’s my person who I go to. So*, *there have been people like that at the institution.”* FG2: 1252–1254.

and

*“But again*, *they’re the researchers*, *we’re not. I don’t know who’s doing all this research you’re talking about*, *but it’s my suggestion that people sometimes are intimidated to reach out.”* FG4:536–539.

#### Resource allocation

The third theme centered on the resource allocation required to conduct and sustain research capacity. The three main subthemes captured included funding, research hours, and infrastructure. All participants expressed the importance of having access to funding to conduct and further their research and contribute to overall capacity building. There was an acknowledgement and appreciation of available internal research funds provided by the institution; however, they also recognized the inherent challenges in applying for and receiving said funds. The challenges to accessing funding were particularly noted by those with limited research experience, who felt that directions were not clearly communicated, and decisions could be made with more consideration of how such funds could develop researchers.

*“So*, *if there’s a way that we could stratify the funding so that as a beginner you get mentorship as well*, *and if you’re marked as an expert then do your thing and have check in points where you’re funding can be pulled.”* FG2:587–593.

Faculty with no research workload were particularly critical of the funding award process, especially when such funding could be explicitly linked to their application for research hours. This was especially evident among participants with interest in developing a program of research that required ongoing application for funded research hours. This need to repeatedly apply for funding was perceived as a source of frustration, ongoing angst and a significant deterrent to doing research, as noted by an administrator:

*“What you want to do is have secure positions*, *[like] a teaching position….[where] you don’t have to keep applying for it every year to maintain that percentage of full time. But if you take on research*, *it’s always temporary. It only lasts as long as that project lasts. And if you want to continue to be financially supported*, *you better be able to jump on another project and that’s a lot of pressure.”* FG4: 896–902.

Focus groups identified and agreed that the institutional support and resources required to conduct research are available and mostly accessible. Most were complimentary about such support, which ranged from access to library resources to computer and necessary software to laboratory facilities and statistical support. Participants agreed on the appropriateness of available physical facilities to conduct their work, and if deficient, they identified collaborative opportunities to address their particular needs.

*“For me*, *I would say we are provided with a generous amount of equipment. So by and large we have the physical infrastructure to do the work that we want to do. That would probably be #1 for me.”* FG1: 84–86.

However, the focus group participants differed in their perceptions of the extent to which resources were available to support their research. They described the limited to no guidance and assistance in preparing and writing grant applications, proposals and manuscripts, access to graduate and undergraduate students, and research assistants. They opined that this impacted their productivity and time to complete work. For example,

*“Vast majority here are working alone or at least I’m working alone. I don’t have a resident [post-graduate student who can assist with research]; I have a student at the best-case scenario helping out. Well*, *that’s the reality.”* FG3: 863–867.

And

*“I found that most of my research has actually been outside of* CMCC *because it’s been an easier process…. I’ve done some research with [teams] and some other stuff through [university] and they have a really nice*, *streamlined approval process where they can help you write grants. And as such*, *I’ve gotten like 3 or 4 grants with another researcher…. So*, *I’ve just kind of taken a step back and started doing research elsewhere as opposed to at* CMCC.*”* FG2: 671–677.

When probed, all focus groups noted and emphasized the importance of the mentorship of novice faculty. They also stated that making available training materials and modules to inform all aspects of the research process is an important aspect of institutional support. They offered creative suggestions on how to support skills training, in that such materials could be provided online, in person or by other institutions.

*I’ve talked to a few different people about it. I’ve kind of journeyed into it a little bit but you know to have someone there to sort of hold my hand a little bit and guide me along the way would be much appreciated because me trying to find my way right now*, *it’s really leading nowhere.* FG3:435–439.

#### Institutional factors

A fourth theme centered on the institutional factors that influence research culture and capacity, including organizational structure, support and planning. Participants described how the institution’s organizational structure, its day-to-day curricular schedule, and its administrative functions could negatively influence RCC by impacting their available time to devote to their research. In reference to structure, participants coalesced around the expectations of faculty and the reality that, for most, their workload did not include time for research, was teaching intensive and a number were part-time with other personal clinical obligations.

*“But I think the other thing too is it was just assumed that faculty was going to get involved in research in some way as it is in universities. And I think we have to recognize … [and consider] specific kind of nuances of a college like ours*, *where such a high percentage of the faculty are part-time and practice in their downtime as opposed to just being on salary and having more*, *if not time*, *at least the opportunity to work some research into your full-time position.”* FG3: 633–640.

Consequently, some participants felt that department managers could advocate for more research hours by requesting such funds during the institution’s budgeting process, but in not doing so, they perceived that the institution was not supporting research.

*“So*, *if the constraint is the budget but you never ask for more money in the budget*, *then that’s not really the constraint … it’s a bit frustrating. Because*, *again*, *I feel like there’s an expectation that we participate in research*, *but it’s not really truly supported.”* FG1: 315–319.

However, the administrative focus group highlighted the challenge identified by the other focus groups that face faculty regarding the expectation of producing scholarly works despite limited skills and interest. They noted recent steps by the institution to change the requirements for promotion; therefore, recognizing faculty for their teaching and non-research related activities could be more representative of their actual skills and contributions.

*“It’s just not realistic that everyone should do research. Why? You know everybody has strengths. Some are incredible teachers but not researchers. So why force someone or encourage someone? They’re great clinicians. There are great researchers. We all have a different skill set and an interest level. But sometimes I think in the past people felt this pressure to do research. You know*, *what a waste of time and energy.”* FG4:522–527.

and

*“And so*, *my research background also allows me to kind of be able to understand better the quality of research. So*, *when I after attending conferences and reading publications published by* CMCC *faculty*, *I was really impressed with the high quality of research*, *and definitely in my perception* CMCC *is leading in research compared to it some other chiropractic institutions.”* FG4:163–168.

Participants questioned the level of institutional support by extrapolating the level of public recognition of the achievements of faculty in research. They perceived that limited communication on research achievements was an indication of lack of support and recognition, which appeared to influence faculty morale and motivation to continue to pursue research. This was particularly notable among those with limited research experience:

*“And until now I can’t see even my name as a research department member. Even on the* CMCC *website I’m not listed as a research department*, *so like. So*, *this is really not encouraging.”* FG2: 745–747.

Participants also commented on the importance of faculty participating in institutional research planning. Despite administrative attempts to engage faculty in institutional planning processes and incorporate their opinions, participants felt differently at least as it pertains to strategic planning in research. All participants appreciated the opportunity to participate in these focus groups and voice their opinions and offer solutions to advance the research culture and capacity of the institution. This was particularly poignant for those who felt a disconnect between themselves and management:

*“…and then the leadership teams we have*, *it doesn’t matter what level they’re at*, *they’re the ones who seem to be doing the planning and telling us what to do. So*, *it’s still top down.”* (FG2:945–947).

Although communication on matters related to research may be shared throughout the course of the year, the communication may have been perceived as unidirectional, with few opportunities for faculty to express their views:

*“This*, *to my memory when I sort of went back through it*, *is the first time I’ve really been actively involved in a conversation around research at the institution*, *I think.”* FG2: 838–840.

At the open internal research day, the feedback from those present supported the interpretation of the study data, confirming the interpretation of the emergent themes and related categories. The attendees of the open internal research day also provided insights and suggestions to address possible solutions to the challenges raised during the focus group sessions. Examples of possible solutions included developing a research skills program for interested faculty, improving communication to faculty and students about potential opportunities to become involved with ongoing or upcoming research projects, developing research mentorship strategies that recognize the efforts of both mentees and mentors and, finally, developing an institutional knowledge translation strategy to highlight current research and enhance the integration of research findings into the curriculum.

## Discussion

Chiropractic educational institutions, as “centers of advanced learning”, should be integral to increasing research capacity and maturing the profession’s research culture; [10] however, there has been very little effort to understand the research capacity and culture at chiropractic educational institutions since 2010 [12]. We used a sequential mixed methods design to evaluate the research capacity and culture at the CMCC. The quantitative findings demonstrated that attributes at the organization or department level were consistently rated as either moderate or high; however, research skills at an individual level were more variable and influenced by factors such as research workload and the highest research-related academic qualification. The qualitative perspectives expressed by the focus group participants reinforced many of the quantitative findings, with some divergence. The information presented in this study can be used to systematically develop strategies to enhance institutional research culture and capacity. These findings can also serve as a comparator for similar future studies at other chiropractic educational institutions.

The research capacity and culture at CMCC can be evaluated by relating quantitative and qualitative findings from the current study to identified barriers and facilitators for healthcare professionals to engage in research, and even more specifically, chiropractic faculty [12]. We defined research capacity as the skills and abilities of individuals and/or institutions to conduct high-quality research. We defined research culture as the institution’s research behaviors, values, expectations and norms [11,13]. Culture-related facilitators that strengthened research capacity included CMCC’s high level of administrative support for research, encouraging faculty to pursue higher research-related academic credentials and support for disseminating research findings through peer-reviewed publications and presentations. The critical importance of leadership’s attitudes toward research to institutional research culture, particularly the sentiment of “research as core business”, has been highlighted by previous work involving a breadth of healthcare disciplines [7,16,17,38–40]. 

Another suggested strength was the availability of research mentorship; however, accessing and providing incentives for such mentorship and the availability of career pathways in research were not strongly supported facilitators. Theoretically, providing faculty with protected time for research, likely coupled with facilitating mentorship from experienced researchers, could be a useful approach for developing career pathways in research and enhancing research capacity [16,38,39,41]. Quantitative results from our survey support a possible positive relationship between having a research workload and a faculty member’s confidence in their own research-related skills. One caveat to this interpretation is the possibility that faculty were provided a workload component for conducting research because of their advanced research-related skills.

Lower scores for survey items surrounding mentorship, research planning and ensuring faculty research career pathways, as well as the identified workload and time-related barriers for engaging in research, were supported by each of the four themes defined by our qualitative analysis. For example, the availability of incentives for mentoring activities was the lowest rated item at the department level for the RCC tool, and the perceived absence of these incentives was identified by focus group participants under the resource allocation theme. Interestingly, the lowest rating for the availability of mentorship incentives was provided by respondents who had completed a residency program as their highest research-related academic qualification. This group was mainly composed of either full-time clinical faculty or part-time teaching faculty. Their noted rating may be explained by findings during the focus group wherein participants contrasted the higher levels of mentorship and support during their program with the lower levels following graduation. Relatedly, being either a part-time faculty member or having a role as a clinical faculty member has been identified as a barrier to chiropractic faculty members engaging in research [12]. The lower score for ensuring that faculty are involved in planning around research overlapped with the sentiment from the focus groups that faculty should be involved in such planning. The high percentage of “unsure” (∼ 1/5) responses at the organizational and departmental levels could be related to focus group comments about limited communication and faculty involvement in research planning, which was part of the institutional factors theme.

Finally, the focus groups expressed a general agreement around institutional support and the availability and accessibility of resources for conducting research; however, this agreement was not fully supported by survey items focused on the institutional availability of resources for conducting research. The sentiment expressed by participants in our administrative focus group regarding the need to consider the practical implications related to the allocation and deployment of resources (e.g., financial, personnel) should be heeded if developing approaches to define career pathways in research. Nonetheless, the foundations of the CMCC’s strong administrative support and infrastructure, combined with a group of experienced researchers, could assist in future research capacity building among faculty through structured mentorship programs [38]. Examples of initiatives that may help address identified barriers in effort to build research capacity, such as structured programs for mentorship and enhancing faculty research skills, were articulated following an internal presentation of the study’s findings. Future research capacity building efforts may look to the Chiropractic Academy of Research Leadership (CARL) and the Melbourne University Sydney Queensland: Impact (MUSQ: Impact) as examples of structured mentorship programs [8,42]. These programs are specifically aimed at creating networks of researchers and fostering career development for researchers in musculoskeletal health.

### Strengths and limitations

One strength of our study was the use of an explanatory mixed methods design with a quantitative priority. As demonstrated above, an advantage of this approach is using qualitative information to further inform quantitative findings. Another strength was the breadth of representation among focus group participants, which included non-researchers, faculty with and without a research workload, and administrators. Similarly, having both faculty and administration among the study team, spanning diverse research experiences and perspectives, may also be considered a strength; however, the fact that all members of the study team are affiliated with the institution being studied may be considered a limitation. Another strength was the use of a validated tool for evaluating RCC [30]. Bilardi and colleagues noted that most tools used to empirically evaluate research capacity are focused at the individual level, which may restrict studies evaluating research capacity [43]. The RCC tool was chosen not only because of its psychometric properties, but also because of its ability to provide valuable empirical information about research culture and capacity at the institutional, departmental and individual levels. A limitation of our study was a response rate of less than 50%, which may have introduced response bias. This rate could be related to the length of the survey or simply reflect limited engagement by faculty. Finally, the findings of our study represent the research culture and capacity at a single private chiropractic institution in a westernized high-income country. Although there were many similarities between the identified barriers and facilitators and those identified in previous research in other healthcare disciplines, our findings may not be relevant to other academic institutions.

## Conclusion

The quantitative and qualitative information in this study provides a baseline evaluation for the research culture and capacity, including barriers and facilitators, at the CMCC as reported by faculty. This information is important for systematically planning, developing, implementing and evaluating future interventions to enhance research capacity. Ultimately, these efforts are aimed at maturing the research culture of the chiropractic profession.

## Electronic supplementary material

Below is the link to the electronic supplementary material.

Supplementary Material 1

Supplementary Material 2

## Data Availability

The data that support the findings of this study are available from the authors upon reasonable request. Some ethical restrictions may apply to the sharing of data to ensure that participants cannot be identified.
