# Supplementary material for: Assessing research culture and capacity amongst faculty at a North American chiropractic institution: an explanatory mixed methods study
Source: Chiropr Man Therap. 2024 Nov 20;32:35. doi: 10.1186/s12998-024-00558-9 (PMC11580544; doi:10.1186/s12998-024-00558-9)
Supplement: Supplementary file 1 — Supplementary Material 1 [file 12998_2024_558_MOESM1_ESM.docx]

**Good Reporting of A Mixed Methods Study (GRAMMS)**

Title: Assessing Research Culture and Capacity amongst faculty at a North American Chiropractic Institution: An Explanatory Methods Study

| **Guideline** | **Place in Article** |
| --- | --- |
| Describe the justification for using a mixed methods approach to the research question | The justification is described at the beginning of the *Methods* section:  “Mixed methods research allows for the collection and analysis of both qualitative and quantitative data and its integration, drawing on the strengths of both approaches.^24^ In this explanatory design, the quantitative data collection and analysis informed the follow-up qualitative phase.^24^ It was determined *a priori* that the quantitative data would not be sufficient to address the complex and multifaceted issues underpinning research capacity and culture;^25^ therefore, the qualitative data would be used to fill in some of the gaps and potentially explain the survey findings.” |
| Describe the design in terms of the purpose, priority and sequence of methods | As mentioned in the *Introduction*, “the aim of the current investigation was to collect information about the extant research capacity and culture (RCC) at the CMCC and to explore the views, attitudes and experiences of faculty regarding research.”  The purpose of the methods and sequence is again answered in the *Methods* section:  “In this explanatory design, the quantitative data collection and analysis informed the follow-up qualitative phase.^24^ It was determined *a priori* that the quantitative data would not be sufficient to address the complex and multifaceted issues underpinning research capacity and culture;^25^ therefore, the qualitative data would be used to fill in some of the gaps and potentially explain the survey findings.”  Under *Methods: Setting, Participants and Recruitment*, second paragraph, we used a modified tailored design method with reminder emails at 1, 3 and 5 weeks for the survey, utilizing “influencers” and union representation to endorse our survey. We then used the sampling measures listed under question 3.  Additionally, modifications were made to the RCC tool to fit our institution’s specific organizational structure, including a component of the survey which allowed participants to identify their research stream (defined in our institutional strategic plan.) We also pilot tested the survey. Further detail is found in *Data Collection: Quantitative Component*, paragraphs 4 and 5. |
| Describe each method in terms of sampling, data collection and analysis | Per *Data Collection: Quantitative Component*, all faculty at CMCC were invited to participate in a modified (see paragraphs 4 and 5, *Quantitative Component*) 52-item Research Culture and Capacity survey. As discussed in *Methods: Setting, Participants and Recruitment*, paragraph 2, recruitment occurred through a modified tailored design method. The survey administration was cross-sectional, over the summer of 2023. Faculty were unable to fill out the survey again once completed. Data were collected using an online survey platform (*Data Collection: Quantitative Component*, paragraph 1). Identifiers including IP address were stripped, and then handed to two members of the research team (SH and MF, see *Quantitative Component* paragraphs 3 and 4, and *Data Analysis* paragraphs 2 and 3). Quantitative analysis consisted of reporting means and frequencies, and descriptive data.  Per *Methods: Setting, Participants and Recruitment*, paragraphs 3 and 4, we used purposive sampling on survey response data to identify focus group participants, followed by snowball sampling. Maximized variation sampling was used to ensure balances in gender, department, role and duration of employment. A research assistant stratified participants into 3 groups by research involvement, with a separate, fourth group comprised of administrators. Per paragraph 2, *Qualitative Component* an experienced qualitative researcher facilitated the focus groups over Zoom, with a recording assistant. Paragraph 3 describes transcription of the audio recording. For qualitative thematic analysis, see paragraph 4 of *Data Analysis*, where the code book and subsequent coding, theme development were described. |
| Describe where integration has occurred, how it has occurred and who has participated in it | Integration occurred *a priori*, as mentioned in the first paragraph of *Methods* – because we recognized that the quantitative data was necessary to fill in gaps to address multi-faceted and complex issues. In the final sentence of *Data Collection: Quantitative Component* we mention that findings were used to inform the qualitative phase, which is further substantiated in the third paragraph of *Data Analysis*, where stratifications are discussed. SH and MF concluded during quantitative analysis that divisions were evident in the results based on respondent type. Reporting the divisions in the results back to the group influenced CAW, DG, DA and SM when they were considering focus group structure and question generation (as appearing in the first sentence of *Results: Focus Group*). This is further evidenced in *Qualitative Component*, paragraph 2, last sentence.  Per Discussion, paragraph 2, the primary use of quantitative methods allowed us to use subsequent qualitative results to reinforce or contradict the findings of the RCC survey. |
| Describe any limitation of one method associated with the presence of the other method | We present the qualitative results’ ability to contradict quantitative results in *Discussion* paragraphs 1 and 2. Such contradictions may represent inconsistencies and may therefore be seen as limitations.  Qualitative analysis often allowed our team to fill in details, or permitted speculation about the shortcomings of the quantitative methods, such as where we link levels of “unsure” responses with the theme of limited communication and faculty involvement in research planning in *Discussion*, paragraph 2. |
| Describe any insights gained from mixing or integrating methods | As noted above in the limitations box, using a mixed methods design with the quantitative component first allowed for further investigation of themes in the focus groups. We were able to probe further for insights during the focus groups by designing questions and organizing groups by research workload, and opting to have an administrative focus group. For article coverage, see *Results: Stratification by Workload* and *Stratification by Highest Research-Related Academic Qualification*.  We recognized the importance of the process as described by our ability to either reinforce or contradict findings in *Discussion*, paragraph 2. The insights of the team are further represented in the way *Methods*, paragraph 1 is written. |

*O'Cathain A, Murphy E, Nicholl J. The quality of mixed methods studies in health services research. J Health Serv Res Policy. 2008;13(2):92-98.*
