# Supplementary material for: Assessing research culture and capacity amongst faculty at a North American chiropractic institution: an explanatory mixed methods study
Source: Chiropr Man Therap. 2024 Nov 20;32:35. doi: 10.1186/s12998-024-00558-9 (PMC11580544; doi:10.1186/s12998-024-00558-9)
Supplement: Supplementary file 2 — Supplementary Material 2 [file 12998_2024_558_MOESM2_ESM.docx]

**Additional File 2:** Focus Group Interview Guide

**Main Questions for all groups unless otherwise specified.**

**Part A:**

General interest question for all groups.

1. In thinking about some of the research you may have been involved with at CMCC:
2. What were some of your challenges?
3. What did you find supportive?
   1. If support, please describe?
   2. If no support, how could your experience have been improved? What type of support would have helped?

1b. For FG 3: Think about the research that is conducted at CMCC:

- 1. What interests you about the research conducted at CMCC?
  2. How do you envision the role of research in an academic institution?
  3. Do you apply research in your role at CMCC? If so, how – teaching in labs? Small groups? lectures? in clinic?
  4. Are you aware of the research conducted at CMCC? If applicable to your area of teaching, do you apply it in your materials? If so, how? If not, why?
  5. How would CMCC be able to get you more involved in research? What type of participation, if any, would you be willing to consider? How can CMCC support this for you?

Collaboration

1. Answers in the survey suggest that there is an opportunity to improve collaboration when it comes to research. How can we support that?

Probe:

- 1. Within CMCC? Within your department and between departments?
  2. How could we involve different groups of people in research in and outside CMCC?
  3. Who do you think are the stakeholders or users of the research you do (or is conducted at CMCC)?
  4. What does collaboration mean to you?
  5. Who are potential collaborators? (e.g., researchers with expertise in different areas, patients, policy makers, educators, clinicians)

**Part B – Specific survey questions**

1. What are your thoughts about how we go about planning to maintain or advance research at CMCC (e.g., strategic plan, institutional plan, budget plan)?

Probes

- 1. Are you familiar with the planning or how it is done?
  2. How can we help get faculty involved in the planning for research within the department and institution?
  3. How can CMCC ensure the planning is clinically relevant and guided by the research (i.e., what are the logical next steps)?
  4. Are you familiar with the research streams? (*For interviewer, streams: biological basis of msk injury and manual therapies; clinical and health services research; education in healthcare; health and wellness; knowledge translation and health policy)*
  5. Are they relevant to your work? Are they important for planning?

1. Are you aware that research can be tied to performance evaluations and promotion at CMCC? What do you think about that? Is that helpful with promoting research capacity and culture? Why/why not?
   1. How have you been approached at your performance review?
   2. Has there been any discussion with respect to getting you involved/interested in research?
2. In the survey people felt that a lot of improvement was needed with mentorship at CMCC. Do you have any suggestions to improve mentorship within your department and within the institution?

Probe:

- 1. What are some of the challenges that you see with respect to improving mentoring?

1. Are there research skills that you would like to improve upon or develop to improve your ability to do research at CMCC? Consider everything from finding literature, designing a study, formulating a question, writing a grant proposal.
   1. How can CMCC improve your experience in these areas?
   2. What kind of advice should the institution and departments give to less experienced researchers?
2. Aside from actually carrying out the research studies, how else would you use research in your day-to-day work?
   1. Does research inform your teaching? How? Why/why not?
   2. Are there any skills that you would like to improve upon to better use research in your day-to-day work at CMCC?

4b. If FG 3 (not interested/low interest in research):

- 1. Is there a reason you have not engaged in research at CMCC? If yes, please provide those reasons. If no, how what might be your hesitation? How can CMCC (department or institution) help facilitate this?
  2. Do you use the evidence that comes from research? Why/why not? How so?
  3. Are you interested in supporting a research culture in other ways? Why/why not? E.g. sharing findings with colleagues, encouraging/supporting others doing research, speaking positively about research, etc.

**Part C: Barriers and motivators**

*Barriers*

1. The survey identified a number of barriers for doing research. What are some of the main barriers for you?

Probes:

- 1. Barriers that came out in the survey: “*lack of time*”, “lack of funds”; “*other work roles that take priority*” and “*the desire for work/life balance*”. What do these mean to you?
  2. Do you have any recommendations to address these barriers?

Probes:

1. What support do you need to further your research interests/work?

e.g., Learn how to develop a question? Writing a research ethics board proposal or grant?? identifying funding agency? How to manage a project? Reporting of grant monies received?

*Motivators*

1. Respondents of the survey noted a number of potential personal motivators for being involved in research. What are some of your motivations and why?

Probes:

- 1. Motivators that came out of the survey: “*to keep the brain stimulated*”, “*increased job satisfaction*” and “*career advancement*”. What do these mean to you?
  2. Survey respondents indicated they were motivated to “*develop skills*”. How can CMCC help with this?

1. FG 3: This group has indicated some hesitation with being involved with research. Why?

Is there anything that would motivate you or help change your mind? Why/why not?

**Part D: End questions**

General questions for the various FG

1. For FG 1: From an institutional and departmental level, what do you need to sustain your engagement in research?
2. For FG 2: What would motivate you to go beyond what you are doing currently and be more involved in research? What would motivate you to fully complete a project from beginning to end as a primary investigator/co-investigator?
3. For FG3: Is there any part of being involved with research that interests you? Why/why not?
   1. If you are not interested in being involved in doing the research, are you willing to support CMCC being a research culture in other ways? How? Why/why not?

E.g., other ways you can support: use evidence in your teaching, talk positively about research, encourage researchers and young investigators, share research with your colleagues, get to know the researchers, etc.
